# The Red Cross and the Nurse

**Published:** 1919-04-19

**Authors:** 


### THE RED CROSS AND THE NURSE.

Born of the war are a comradeship and a brotherhood, national and international, such as never before existed. Distinctions of class and creed and race will probably never be obliterated, but they have ceased to be dominant. Rich and poor, men of high rank and humble, of every race and colour have fought and died together for a common cause—the cause of humanity. And while the battle raged the people in the homeland watched and waited in sorrow and in hope—as women watch on the shore when their sons and husbands are in the storm.

To my mind the common bond of the nations has been and is the Red Cross, the Cross of Healing. We have not yet lived long enough to see the perspective of things, and this truth is not therefore realised or appreciated. A century, or even a generation, hence it will be apparent that this mystic symbol stood in the blackest hours of the war for the world's salvation and regeneration. It sanctified and ennobled the war worker's efforts during the conflict, and when hostilities ceased, as though guided by a power unseen, the world's Red Cross representatives gathered together in council with the object of co-operating for the benefit of humanity. Have you noticed what is happening at Cannes? A great Red Cross Congress is in progress with the object of forming a world co-operation against disease of every kind, and providing a means of relief in emergencies such as fire, famine, and pestilence. Those who rallied round the Red Cross during the war will be requisitioned for service in the aftermath. Geneva, it is hoped, will become the central bureau of information and activity. Information relating to research, investigation, public health, sanitation, maternal and child welfare, legislative reform will be received at Geneva, and thence issued to every Red Cross organisation in the world. The idea is magnificent, and, so far as I am able to ascertain, finds its origin and champion in Mr. Henry P. Davison of the American Red Cross. It is a great conception with infinite possibilities. As Sir Arthur Lawley said at Cannes: "If only we can rise to a great occasion, we may yet lead the stricken peoples of a distracted world through the present dark night of sorrow to the golden dawning of a better day."

All this has a special message for the British nurse. It has a special significance for the College of Nursing, which is without doubt a child of the Red Cross. The trained nurse, indeed, although she may have forgotten her origin, is also a child of the Red Cross, a fact which certain dames who have uttered many unworthy sentiments concerning V.A.D.s would do well to bear in mind. There is no doubt that one of the persons on whose efficient and effectual service the whole fabric of health reform, national and international, depends is the trained nurse. This is so obvious that the fact merely requires to be chronicled. But what is less apparent, perhaps, is that the necessary supply of such nurses is wholly inadequate, and that public attention will have to be drawn to this. The nation needs nurses to-day just as yesterday it needed munitions, and they will have to be manufactured and employed. Not in hospitals only, but in the homes, the workrooms, the schools, are their services required. And this applies as much to Britain beyond the seas as to the little group of islands that we call home. It is here that the College will find the practical utility of its imperial ideal; here, also, it will have an opportunity of making the ideal real.

Nurses! more nurses!! better nurses!!! the necessity for them must be dinned into the ears of the public until all the people realise. The standard of employment and of education must be raised, and the profession made worthy of a great nation. England has until now earned and enjoyed the reputation of being the pioneer of civilisation in every part of the world. In the nursing department there is a danger of her lagging behind, and to the College of Nursing the country will undoubtedly look for stimulation and leading. The British public is practically uninformed as to the vital national and individual requirements in health matters. Hence the necessity for vigorous and unceasing propagation.

IERNE.

